# Unraveling participant motivation dynamics in local-centric secondhand digital sharing platforms

**DOI:** 10.1371/journal.pone.0337603

**Published:** 2025-12-26

**Authors:** Hana Kim, Gyuhwan Kim, Taehwa Lee, Joohee Lee

**Affiliations:** 1 School of Digital Humanities & Computational Social Science, KAIST, Daejeon, South Korea; 2 Korea Employment Information Service, Chungcheongbuk-do, South Korea; 3 Department of Urban Administration, University of Seoul, Seoul, South Korea; 4 Department of Climate and Energy, Sejong University, Seoul, South Korea; Gannon University, UNITED STATES OF AMERICA

## Abstract

This study investigates the motivational and attitudinal drivers of user engagement in local-centric digital sharing platforms (LDSPs), with a focus on secondhand consumption through Karrot Market in South Korea. Grounded in Self-Determination Theory and the Theory of Reasoned Action, we examine how economic, environmental, interaction, and reputation motivations, together with attitudes and socioeconomic characteristics, influence individuals’ behavioral intentions to purchase secondhand goods. Using structural equation modeling (SEM) on survey data from 449 Seoul residents with prior Karrot use experience, we found that economic motivations were the strongest direct predictor of both attitudes (i.e., positive stance toward buying secondhand on Karrot) and behavioral intentions (i.e., intention to buy secondhand on Karrot). Reputation motivation also exhibited a direct effect on behavioral intention, while environmental and interaction motivations showed influences on behavior only indirectly through attitudes. Multigroup SEM further revealed that these pathways vary by age and marital status, indicating heterogeneous motivational patterns across user segments. Environmental motivation was more salient among users in their 20s and 50s, while reputation held greater importance for older users. Our findings highlight the central role of attitudes as mediators in translating relatively more intrinsic motivations, particularly for environmental and social drivers, into behaviors. Taken together, we offer practical insights for platform design and policy interventions, including strategies to visualize environmental impact, incentivize sustainable behaviors, and tailor engagement approaches by user characteristics. By decoding the multidimensional motivations behind LDSP participation, this research contributes to emerging scholarship on local-centric secondhand commerce and informs efforts to advance community-based circular consumption.

## 1. Introduction

Cities are often regarded as both epicenters of sustainability challenges and incubators of innovative solutions [1]. The current unsustainability of urban metabolism can be attributed to several factors, with large-scale consumption and resulting waste playing central roles [2,3]. The level of material demands generated by urban lifestyles has been met primarily through a linear economy, characterized by intense resource use and waste disposal issues [4].

As a counter model, the circular economy emerged to restore urban material sustainability and encourage innovative approaches in both production and consumption [5–10]. Achieving sustainable consumption requires systemic change and society-wide, inclusive engagement, with citizens playing a vital role as agents of this transformation [11–12]. Indeed, consumers hold substantial leverage to close the resource loop and drive sustainability transitions from the ground up [13].

Citizens can engage in sustainable consumption behaviors via different channels, especially through digital sharing platforms (DSPs) [14]. DSPs cover diverse items and services, including accommodation (e.g., Airbnb), car and ride-sharing (e.g., Zipcar), peer-to-peer (P2P) employment (e.g., PeoplePerHour), and peer-to-peer resource sharing (e.g., Peerby) [15]. Secondhand DSPs allow users to access a wide range of secondary goods and detailed information about their conditions, addressing users’ reservations about trading pre-owned items and reducing waste [14,16–20]. Through these platforms, consumers can contribute to sustainable consumption and the circular economy by extending the lifetime and utilization of everyday items [21].

More recent DSPs have experimented with local-only trading and user interactions, demonstrating innovative approaches to digital, neighborhood-based commerce. These local-centric DSPs (hereafter LDSPs) aim to foster digitally mediated social interactions and the exchange of material or non-material resources exclusively within the neighborhood.

Understanding the motivations of individual engagement in the purchase of secondhand items through LDSPs is important for promoting and expanding engagement. In addition, the motivational dynamics behind DSP-based secondhand trading that involves ownership transfers have been underexplored, especially in non-Western contexts. Furthermore, there has been limited empirical research on LDSPs, which emphasizes localness in sharing activities and the resulting effects.

To address these gaps, the present study focuses on four key motivations—economic, environmental, interaction, and reputation—which capture both intrinsic and extrinsic factors commonly associated with DSP participation. By specifying these distinct motivational drivers, this study seeks to provide a more nuanced understanding of their influence on attitudes and behavioral intentions toward secondhand trading through LDSPs through the lenses of self-determination theory (SDT) and theory of reasoned action (TRA). Specifically, this study addresses the following research questions: How do these motivations influence individuals’ behavioral intentions to purchase secondhand goods through DSPs? How do economic, environmental, interaction, and reputation motivations influence individuals’ attitudes toward purchasing secondhand goods through DSPs? Furthermore, how do these relationships vary across user groups with different socioeconomic characteristics and prior experiences with DSPs?

Addressing these questions holds both theoretical and practical significance. Theoretically, this study extends the application of the TRA to LDSP-based secondhand trading and by incorporating a broader set of motivational drivers—economic, environmental, interaction, and reputation motivations, which are categorized in intrinsic and extrinsic motivations in SDT. Moreover, by simultaneously investigating the moderating effects of socioeconomic characteristics and DSP experience, this study proposes a comprehensive and context-specific model of consumer behavior in non-Western settings. Practically, the findings offer insights for DSP platform managers and policymakers to design targeted strategies that promote sustainable secondhand consumption, tailored to diverse user groups.

For analysis, we use the case of *Karrot Market*, also known as Danggeun Market, a leading LDSP in South Korea. This mobile app-based DSP, widely used in the country, is primarily designed to revitalize local communities and economies by facilitating the trade and donation of used items among residents. Karrot Market operates within predefined local boundaries. We employed structural equation modeling (SEM) using online survey data from 450 Seoul citizens who had used Karrot Market.

## 2. Theoretical background and literature review

### 2.1. The effects of intrinsic and extrinsic motivations

SDT provides a useful framework for understanding why individuals engage in certain behaviors [22]. It conceptualizes motivation along a continuum from less to more self-determined forms: specifically, from amotivation (lack of intention to act), through extrinsic motivation (driven by external rewards or pressures), to intrinsic motivation (driven by inherent enjoyment or meaning). SDT also explains how external motivations can be internalized and integrated with personal values, leading to more genuine and lasting behavioral engagement, such as sustainable consumption. While our study does not empirically examine this internalization process, it applies SDT to categorize and analyze the motivations influencing users’ pro-environmental attitudes and behaviors on LDSPs.

Building on prior research identifying economic, environmental, and social motivations as common drivers of DSP use [20,23–31], we draw particularly on the analytical framework used in Hamari et al. [26] to classify four types of motivations: economic, environmental, interaction, and reputation motivation. These align with key points along the SDT continuum, offering a theoretically grounded perspective on user engagement in LDSPs like Karrot.

Specifically, *economic motivation* aligns with external regulation, as it is driven by tangible rewards such as cost savings. *Reputation motivation* often reflects introjected regulation, driven by internal pressures to maintain self-worth or gain social approval. *Environmental motivation* aligns with identified regulation, where users value sustainability and integrate it into their personal beliefs, even if behavior is not inherently enjoyable. *Interaction motivation*, involving enjoyment of social connections, is closest to intrinsic motivation, where participation is pursued for its inherent satisfaction. Together, these four motivations span the SDT continuum from controlled to autonomous forms, providing a comprehensive lens to examine behavioral diversity among DSP users.

Many studies have consistently identified economic motivations as strong predictors of DSP use. Hamari et al. [26] and Vaclavik et al. [31] found that economic motivations were decisive for user participation in DSPs, while Böcker and Meelen [23] highlighted their role in high-cost sharing contexts, like accommodations. Although rooted in controlled forms, economic motivations may still influence users’ pro-environmental attitudes and behaviors.

The influence of environmental motivations has been more variable. Ek Styvén and Mariani [25] found that sustainability concerns significantly motivated secondhand clothing purchases through DSPs, and Böcker and Meelen [23] reported similar findings in car and ride sharing. However, Hamari et al. [26] and Piscicelli et al. [29] found that sustainability concerns had limited effects, especially in access-based services. SDT suggests these mixed findings may stem from varying degrees of internalization and alignment with personal values.

Interaction motivation has been examined as a social driver. DSPs are known for enabling social interactions similar to offline shopping [24], especially in P2P transactions requiring communication to negotiate schedules, prices, or transaction details [19]. LDSPs like Karrot can further enhance these opportunities through offline encounters. Böcker and Meelen [23] found that social motivations were pronounced in accommodation- and meal-sharing sectors. Lho et al. [32] showed that social interactions shape users’ sense of social presence and continued participation.

Reputation motivation, while related to social interaction, focuses specifically on how others evaluate users’ behavior. Trust is a critical factor in DSPs [27], and many platforms disclose reputation scores on social qualities like friendliness, politeness, and punctuality [31,33]. Karrot gamifies the reputation system using ‘manner temperature’ system. Hamari et al. [26] and Yildiz and Altan [34] found that reputation was not a significant motivator. Thus, we examine whether the enjoyment of managing one’s reputation in DSPs influences pro-environmental attitudes and behaviors.

### 2.2. The role of attitudes in shaping pro-environmental behaviors

Predicting user participation in DSPs requires comprehending not only their motivations for purchasing items through DSPs but also their attitudes toward behavior [25,26,30]. Our model, therefore, includes attitude as an important precursor of behavioral intentions, drawing on TRA [35], which considers attitude as a key factor driving behavioral intentions. TRA, a foundational framework in social psychology, posits that an individual’s behavior is primarily determined by their behavioral intention, which is influenced by two key factors: attitude toward the behavior and subjective norms. This theory, along with its extension, the Theory of Planned Behavior (TPB) [36], has been extensively applied to understanding and predicting pro-environmental behaviors.

Recent research has consistently demonstrated that positive attitudes toward sustainable consumption are associated with stronger behavioral intentions and actual pro-environmental behaviors. For example, Liobikienė et al. [37] found that Lithuanian consumers with favorable attitudes toward environmentally friendly products were significantly more likely to engage in green purchasing behavior. Similarly, Prakash and Pathak [38] demonstrated TPB that attitudes toward eco-friendly products strongly predicted green purchase intentions among young consumers in India. Yadav and Pathak [39] found that attitudes significantly influenced intentions to purchase green products, while Paul et al. [40] reported that positive attitudes toward sustainable products had a strong effect on purchase intentions among consumers. These studies collectively support the inclusion of attitude as a key variable in our model to better understand how diverse motivations translate into pro-environmental behavioral intentions within DSP contexts.

### 2.3. The moderating role of socioeconomic characteristics

Previous research suggests that the effects of motivations on attitudes and behaviors in DSP contexts can vary depending on individual socioeconomic characteristics such as age, gender, income, and education level. For example, economic motivations have been shown to vary across demographic groups. Böcker & Meelen [23] found that economic motivations were stronger among younger and lower-income groups when they were buying and selling shared assets. Gender differences have also been observed, with women showing a stronger association between economic motivations and behavioral intentions regarding secondhand clothing purchases [25].

Similarly, environmental motivations have been found to differ across gender and age groups. Böcker and Meelen [23] reported that women tend to exhibit stronger environmental motivations for using DSPs, while younger individuals generally demonstrate greater concern for environmental sustainability compared to older generations. Laurenti and Acuña [27] confirmed this pattern among student users. However, findings across similar studies are not always consistent; Ek Styvén and Mariani [25] found cases where age-related differences in environmental motivations were minimal or even reversed.

Social motivations can also be influenced by socioeconomic characteristics. Böcker and Meelen [23] observed that younger DSP users and those with high income or educational attainment exhibited lower social motivations compared to other groups, suggesting that social aspects may play a more significant role for older or less affluent users.

Building on these studies, our research model investigates how socioeconomic characteristics moderate the relationship between motivations, attitudes, and behavioral intentions in LDSP contexts. This approach allows for a more nuanced understanding of user engagement and pro-environmental behaviors across different social groups.

### 2.4. Shared gaps in the literature and this study’s contributions

This section synthesizes the key papers discussed in the previous sections to identify shared research gaps by highlighting both the commonalities and recurring limitations among them. Through this synthesis, we demonstrate how these existing studies inform and guide our research focus and design. Based on this review, we clarify the contributions the present research seeks to make.

A key commonality across prior research on DSPs and related systems is the increasing attention to various types of relatively more intrinsic motivations—such as sustainability, social interaction, enjoyment, and trust—as drivers of user participation or participation intention. Especially, many studies highlighted the social and relational dimensions of user motivations as an underexplored area within the field [27,31,41]. In doing so, economic motives were commonly included not only as key independent variables but also as benchmarks for evaluating the explanatory power of alternative motivations beyond economic gain. Previous studies employed a variety of theoretical frameworks from psychological and behavioral studies, ranging from SDT and TPB to value-based adoption models (e.g., [29,32]). A summary of the key studies reviewed is presented in S1 Table.

Despite the meaningful contributions from the prior studies, key gaps remain in the limited understanding of how diverse motivations and user characteristics shape pro-environmental attitudes and behaviors within these platforms. Specifically, this study addresses the following three gaps. First, local-centric forms of DSPs have been underexplored in previous research. Most existing research has primarily examined motivations for using generalized or sector-specific sharing platforms—such as accommodation, transportation, or clothing—typically operating at regional, national, or global scales rather than the local level. While these studies have clarified how particular motivations function across different contexts, limited attention has been paid to the case of DSPs grounded in local, in-person interactions, such as Karrot, which introduce distinct behavioral and social dynamics by combining offline exchanges with neighborhood-based digital interfaces.

Moreover, while economic motivation is frequently confirmed as a key driver, findings on environmental and social motivations remain mixed and inconclusive, with some studies identifying strong effects (e.g., [23,25,27]) and others reporting minimal influence (e.g., [26,29,31]). This inconsistency suggests a need to examine these motivations within broader theoretical frameworks that account for the continuum of extrinsic and intrinsic motivations, as posited by SDT. The unclear role of environmental and social motivations observed in previous studies indicates the need for similar investigations tailored to LDSPs.

Also, a limited number of studies have explored how users’ socioeconomic characteristics interact with motivational factors in shaping attitudes and behavioral intentions to use DSPs. While demographic patterns such as age, gender, and income have been examined in previous studies, few provides fine-grained analysis of localized platforms like Karrot Market that blend digital and physical community interactions.

## 3. Methods

### 3.1. An overview of Karrot Market

In South Korea, many DSPs facilitating P2P trades have become popular among those looking for used goods. According to a consumer survey in the second half of 2022 conducted among mobile phone platform users aged 14 or older, 60% of Koreans bought or sold used goods through DSPs [42].

Among the top three platforms, Karrot’s share is dominant in the DSPs (87%, considering multiple-choice responses). Since its launch in 2015, Karrot Market has seen rapid success; the number of monthly active users registered reached about 18 million in August 2022 [43]. Danggeun, the Korean name of Karrot, is an abbreviation for “near you” in Korean that is pronounced the same as “carrot” in Korean [44], so it is branded to be easily remembered. As its name indicates, the platform is designed to allow users to find secondhand products only locally, based on where they live [45]. The platform was launched in the U.S., Canada, Japan, and the UK [44].

Buying secondhand goods online is accompanied by high uncertainty and risk of fraud, as discussed in [46]. Unlike other platforms, selling and buying products in Karrot is limited to neighbors within 10 km [45], which is credited as the main factor for its rapid growth. Only individuals with location-based authentication can see postings and sell their products through this platform. People generally buy or sell their products through in-person trading rather than through the postal service. Afterward, the platform allows the buyer and the seller to evaluate each other’s promptness and politeness. These scores are expressed and accumulated as a ‘manner temperature,’ which is meant to help address buyers’ uncertainties and enhance trust in the exchanges.

Through its local-centric design and enhanced transparency, this unique platform has alleviated the challenges that users often experience in P2P trades [47]. Beyond P2P trades, the platform functions as a community social media by sharing local information such as festivals, lost and found, and quality restaurants. In terms of its operating mechanism and mission, Karrot aligns with ownership transfer models of DSPs [26] and social interaction-oriented DSP markets [19].

### 3.2. Research model and hypotheses

**Fig 1:**
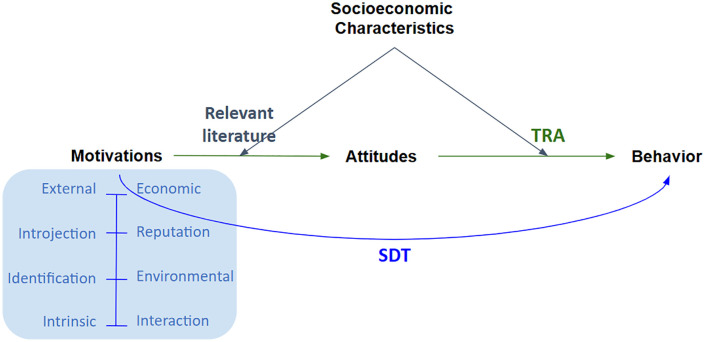
Theoretical framework based on SDT and TRA.

Fig 1 presents the theoretical foundation of this study, which integrates SDT and TRA to explain individual engagement with LDSPs, specifically in the context of secondhand goods transactions. The framework begins with four key user motivations—economic, reputation, environmental, and interaction—which are grounded in SDT. This categorization reflects the extent to which each motivation is internalized and autonomous in the context of LDSP use. In line with TRA, attitudes are positioned as a mediating variable, transmitting the effects of motivation to behavioral intention. By integrating SDT and TRA, our model not only categorizes motivational factors along a self-determination continuum but also theorizes how these motivations lead to secondhand trading on LDSPs —either directly or indirectly through attitudes. Furthermore, we account for the moderating effects of socioeconomic characteristics (e.g., age, marital status), which may condition the strength or direction of these relationships across different user segments.

Based on this framework, the hypotheses for this research are as follows:

- H1a-d: Economic (a), environmental (b), interaction (c), and reputation (d) motivations influence positive attitudes toward the purchase of secondhand goods through the DSP.
- H2a-d: Economic (a), environmental (b), interaction (c), and reputation (d) motivations positively influence behavioral intentions to purchase secondhand goods through the DSP.
- H3: Positive attitudes influence behavioral intentions to purchase secondhand goods through the DSP.
- H4-13: Personal characteristics (age, income, gender, education, and prior DSP experience) moderate the association between motivations, attitudes, and behaviors.

**Fig 2:**
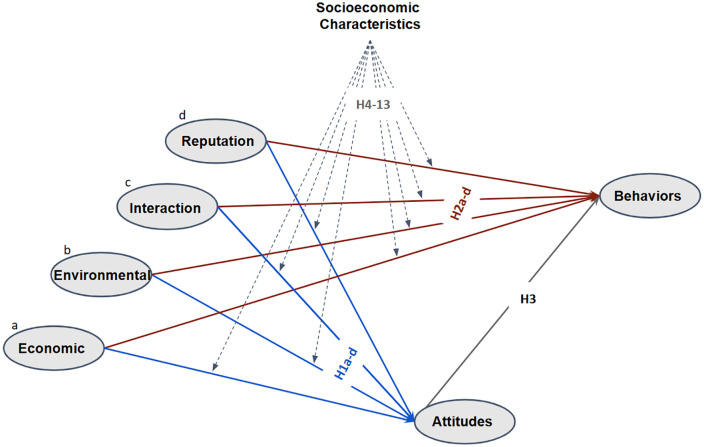
Research model and hypothesis. Notes. The conceptual model is adopted from [25,27,30]. Four motivation variables (economic, environmental, interaction, and reputation) can affect behavior directly and indirectly through attitude. Socioeconomic characteristics refer to age, income, gender, education, and prior DSP experience. Hypotheses H4–H8 test the moderating effects of these socioeconomic characteristics on the relationship between motivations and attitudes, while H9–H13 test their moderating effects on the relationship between motivations and behaviors.

Fig 2 depicts our conceptual model and its hypotheses.

### 3.3. Data collection and measurement

We conducted a structured online survey with 56 questions, including demographic information (see Table 1 for key questions; see S1 File for the full questionnaire). Responses for motivation, attitude, and behavior variables were measured using Likert-scale questionnaires. The responses to the survey were collected from Seoul residents, taking into consideration their demographic profile (age, gender, and income levels). Participants were recruited through Macromill Embrain, a survey company that owns South Korea’s largest online panel group (about 1.7 million individuals). Participants had to be older than 19 and have experience buying products in Karrot. This study was approved by the KAIST Institutional Review Board (KH2022−119), which granted a waiver of written consent as no personal information was collected from the respondents. After a pilot survey with 35 people, we conducted the main survey from August 22–25, 2022, with 300 people, and from September 8–13, 2022, with 115 people living in Seoul. The pilot survey was conducted to evaluate the clarity, wording, and logical flow of the questionnaire. No significant issues were identified during the pilot test. The process helped confirm that respondents clearly understood the questionnaire items and did not require revision. Based on this confirmation, the same questionnaire was used for the primary survey.

**Table 1:** Constructs and questions used in the survey.

Constructs	Indicators	References
Economic motivations	eco1: I want to save money. eco2: I don’t want to pay more just because an item is new. eco3: I want to buy items at a fair price.	Adapted by the authors from [25]
Environmental motivations	env1: I want to reduce resource consumption (related to the production and sale of goods) by buying fewer new products. env2: I want to reduce energy consumption (related to the production and sale of goods) by buying fewer new products. env3: I want to reduce waste (such as plastic and packaging) by buying fewer new products. env4: I want to protect the environment by buying fewer new products. env5: I want to practice sustainable consumption by buying fewer new products.	Adapted by the authors from [25] and [26]
Interaction motivations	int1: It is fun to communicate (chat) with local neighbors while buying used items on Karrot. int2: I am interested in communicating (chatting) with local neighbors while buying used items on Karrot. int3: I enjoy communicating (chatting) with local neighbors while buying used items on Karrot.	Adapted by the authors from [26]
Reputation motivations	rep1: I am interested in managing my ‘manner temperature’ (reputation score) while buying used items on Karrot. rep2: I enjoy managing my ‘manner temperature’ (reputation score) while buying used items on Karrot.	Constructed by the authors – Please refer to this study.
Attitude	att1: I find buying used items on Karrot convenient. att2: Buying used items on Karrot is safe. att3: Buying used items on Karrot feels reliable. att4: Buying used items on Karrot is a wise decision.	Adapted by the authors from [25]
Behavior	beh1:I intend to continue to buy used items on Karrot frequently. beh2: If possible, I intend to buy used items on Karrot frequently. beh3: I am likely to buy used items on Karrot frequently.	Adapted by the authors – with consideration of [25] and [50]

*Note*. Given that direct translation of survey items from selected references may result in unnatural phrasing in Korean or may fail to convey the original intent, such an approach could compromise the validity and reliability of responses [51]. Following the practice of [30], the authors of this study (three experts in environmental policy and one expert in survey methodology) jointly reviewed the items. We found that many of the original English items would sound unnatural in Korean, unless properly contextualized, and thus revised them with careful attention to readability and interpretability in the Korean context. The full survey questionnaire is available in S1 File.

To ensure data quality, a Z-score analysis was conducted on all latent constructs. For each respondent, average scores for items within each latent variable were calculated and standardized. Extreme cases (top and bottom 0.05%) based on the Z-score distribution were identified as outliers and excluded from the analysis. As a result, one out of 450 responses was excluded, yielding a final dataset of 449 valid responses. This sample size is considered adequate for SEM, as previous studies suggest that a minimum of 200 cases is generally required to ensure reliable estimation and model fit [48,49]. The distribution and box plots of Z-scores for each latent variable are provided in S1 Fig and S2 Fig.

Among respondents, 209 were men (45.7%) and 240 were women, with an age range of 20–68 years (mean age = 39.21). The age distribution was as follows: 20–29 years (18.7%, n = 84), 30–39 years (33.1%, n = 148), 40–49 years (28.7%, n = 129), and 50 or older (19.6%, n = 88). Regarding monthly income, 6.4% earned less than 1 million KRW (n = 29), 8.0% earned 1–2 million KRW (n = 36), 12.2% earned 2–3 million KRW (n = 55), 15.1% earned 3–4 million KRW (n = 68), 14.4% earned 4–5 million KRW (n = 65), and 43.6% earned more than 5 million KRW (n = 196). For education level, 75.6% had attended or graduated from college (n = 340), 13.3% held a master’s degree or higher (n = 60), 10.0% were high school graduates (n = 44), and 1.1% had less than a high school education (n = 5). Additionally, 31.3% (n = 140) reported using DSPs other than Karrot, while 68.7% (n = 309) had not. A histogram illustrating the distribution of these characteristics is provided in S3 Fig.

## 4. Results

### 4.1. Measurement model results

To determine the underlying structure of the measurement items, an exploratory factor analysis (EFA) was conducted prior to confirmatory factor analysis (CFA). The Kaiser-Meyer-Olkin (KMO) measure verified the sampling adequacy (KMO = 0.87), and Bartlett’s test of sphericity was significant (p < 0.001), indicating that the correlations among items were sufficiently significant for EFA. Based on the Kaiser criterion (eigenvalues > 1) and inspection of the scree plot, a five-factor solution was extracted. Varimax rotation was applied to improve the interpretability. Detailed EFA results, including the scree plot and factor loadings, are presented in S4 Fig and S5 Fig. To assess the possibility of common method bias (CMB), we conducted Harman’s single-factor test, which showed that the first factor accounted for only 29.2% of the total variance, suggesting that CMB is unlikely to pose a serious concern.

CFA was conducted to evaluate the reliability and validity of the latent constructs within the measurement model, which includes economic, environmental, interaction, and reputation motivations, as well as attitudes and behavioral intentions (see Table 2). Reliability analyses indicated satisfactory internal consistency for all constructs, with Cronbach’s α and composite reliability values exceeding 0.70.

**Table 2:** Results of confirmatory factor analysis.

	Mean (std)	Factor loading	95% C.I.	Z-statistic	Cronbach’s α	CR	AVE
Economic motivations							
eco1 eco2 eco3	5.85 (1.05) 5.42 (1.32) 5.69 (1.02)	0.716 0.661 0.778	0.654–0.778 0.594–0.728 0.721–0.835	22.723 19.435 26.749	0.744	0.763	0.519
Environmental motivations							
env1 env2 env3 env4 env5	5.21 (1.41) 5.00 (1.44) 5.04 (1.52) 4.99 (1.53) 5.21 (1.34)	0.838 0.862 0.860 0.910 0.837	0.801–0.875 0.831–0.893 0.827–0.893 0.884–0.937 0.804–0.870	44.274 53.958 51.375 66.690 49.614	0.939	0.935	0.743
Interaction motivations							
int1 int2 int3	4.09 (1.53) 3.84 (1.61) 3.86 (1.58)	0.887 0.939 0.946	0.864–0.910 0.923–0.955 0.931–0.961	76.362 116.531 123.657	0.946	0.946	0.855
Reputation motivations							
rep1 rep2	4.33 (1.52) 4.24 (1.50)	0.950 0.933	0.924–0.976 0.906–0.960	71.142 67.757	0.950	0.940	0.886
Attitude							
att1 att2 att3 att4	5.18 (1.07) 4.67 (1.11) 4.61 (1.12) 5.16 (1.00)	0.604 0.739 0.784 0.721	0.536–0.672 0.686–0.792 0.736–0.832 0.666–0.776	17.421 27.375 31.987 25.700	0.798	0.806	0.511
Behavior							
beh1 beh2 beh3	5.69 (0.96) 5.17 (1.15) 5.12 (1.17)	0.776 0.894 0.854	0.732–0.819 0.864–0.924 0.820–0.888	34.980 58.399 49.073	0.875	0.880	0.710

*Note.* χ2 = 496.021 (df = 152, *p* < 0.001), GFI = 0.897, AGF I = 0.857, CFI = 0.949, TLI = 0.936, RMSEA = 0.071 [95%, 0.064–0.078], SRMS = 0.056. The fit indices of the measurement model were obtained by checking the MI values and setting the covariances among the measurement variables (env3 and env4, env1 and env2, and env1 and env4).

To improve model fit, modification indices (MI) were examined, and covariances between measurement errors were allowed where theoretically justified and MI values exceeded 10. These adjustments enhanced overall fit indices to acceptable levels, supporting the robustness of the measurement model for subsequent structural analysis.

The factor loadings for most observed variables exceeded the commonly accepted threshold of 0.70, indicating a strong positive correlation and confirming that these observed variables were reliable indicators of the underlying latent constructs at a statistical significance level of .001. Although 0.70 is the standard threshold, loadings above 0.60 are often deemed acceptable in SEM [52,53]. Retaining these loadings did not significantly impair the overall fit of the measurement model.

Cronbach’s α for all variables was 0.70 or higher, indicating robust internal consistency among the questionnaire items [54]. The composite reliability (CR) for each construct also surpassed the 0.70 threshold, further supporting the reliability of the measurement model [55]. The average variance extracted (AVE) values for each latent variable also substantiated the model’s reliability, with an AVE value of 0.50 or higher considered acceptable, indicating the construct explains more than half of the variance in observed variables [56]. Following Fornell and Larcker’s method [57], we examined whether the square roots of the AVEs of the latent variables were greater than their intercorrelations, ensuring the distinctiveness of each variable. The discriminant validity for each concept was confirmed (See the results in S2 Table).

### 4.2. Structural model results

Table 3 summarizes the statistically significant results from our SEM analysis. Specifically, we examined how economic, environmental, interaction, and reputation motivations affect individuals’ attitudes and behavioral intentions, in line with the hypotheses outlined in Section 3.

**Table 3:** Results of structural equation modeling.

		Hypothesis path	β	95% C.I.
Direct effect	H1a	Economic motivations → Attitude	0.452***	0.351–0.553
	H1b	Environmental motivations → Attitude	0.112*	0.010–0.215
	H1c	Interaction motivations → Attitude	0.311***	0.187–0.435
	H1d	Reputation motivations →Attitude	0.112	−0.012–0.236
	H2a	Economic motivations → Behavior	0.341***	0.226–0.456
	H2b	Environmental motivations → Behavior	0.031	−0.060–0.122
	H2c	Interaction motivations → Behavior	−0.085	−0.204–0.034
	H2d	Reputation motivations → Behavior	0.146**	0.035–0.256
	H3a	Attitude → Behavior	0.469***	0.348–0.591
Indirect effect	Economic motivations → Attitude → Behavior		0.212***	0.142–0.282
	Environmental motivations → Attitude → Behavior		0.053*	0.002–0.104
	Interaction motivations → Attitude → Behavior		0.146***	0.075–0.217
	Reputation motivations → Attitude → Behavior		0.052	−0.007–0.112

*Note.* *p < 0.05, **p < 0.01, ***p < 0.001. χ^2^ = 496.021 (df = 152, *p* < 0.001), GFI = 0.986, AGFI = 0.978, CFI = 0.949, TLI = 0.936, RMSEA = 0.071[95%. 0.064–0.078], SRMR = 0.053.

For direct effects, economic motivations exhibited a significant positive influence on attitudes toward purchasing secondhand goods at Karrot (β = 0.452, p < 0.001), supporting H1a. Environmental motivations showed a positive effect on attitudes (β = 0.112, p < 0.05), supporting H1b. Interaction motivations significantly influenced attitudes toward using Karrot (β = 0.311, p < 0.001), confirming H1c. Reputation motivations did not have a statistically significant effect on attitudes, and thus H1d was not supported.

Regarding behavioral intentions, economic motivations exhibited a significant positive direct effect (β = 0.341, p < 0.001), supporting H2a. Reputation motivations also positively influenced behavioral intentions (β = 0.146, p < 0.01), supporting H2d. Environmental and interaction motivations did not show significant direct effects on behavioral intentions, and therefore H2b and H2c were not supported.

Attitudes were found to have a strong positive effect on behavior (β = 0.469, p < 0.001), supporting H3a. This indicates that attitudes play a critical role in mediating the relationship between motivations and behavioral intentions. Our analysis also identified several significant indirect effects. Economic motivations (β = 0.212, p < 0.001), environmental motivations (β = 0.053, p < 0.05), and interaction motivations (β = 0.146, p < 0.001) indirectly influenced behavior through attitude. Reputation motivations did not show a statistically significant indirect effect.

In sum, the results highlight economic motivations as the strongest predictor of both positive attitudes and behavioral intentions toward purchasing secondhand goods through Karrot. Although environmental and interaction motivations did not directly affect behavior, they were found to influence behavior indirectly through attitude.

### 4.3. Multigroup SEM results

To examine whether the structural relationships among latent variables differed across groups, we conducted a multigroup SEM analysis, following a sequential invariance testing procedure [58,59]. The analysis begins with an unconstrained measurement model (UM), where each group’s parameters were independently calculated without equality constraints imposed across groups. Next, we tested for factor loading invariance by specifying a constrained measurement model (CMM), in which factor loadings were set to be equal across groups. Chi-square difference tests between UM and CMM were conducted for each grouping variable. The results showed no significant differences, indicating that the constructs were measured equivalently across groups, thereby justifying the comparison of structural path coefficients. Then, we specified a constrained structural model (CSM) by additionally constraining the structural paths to be equal across groups. Comparing the CMM and CSM models via chi-square difference testing allowed us to evaluate whether the relationships among latent variables significantly differed by group. All model fit statistics and step-by-step comparisons are reported in S3 Table.

The multigroup SEM results revealed several group-specific patterns. In the age-based comparison, environmental motivations significantly influenced behavior among participants in their 20s and 50s, but not in other age groups. Interaction motivations significantly influenced attitudes in the 30s and 40s groups. Reputation motivations significantly affected attitudes in the 40s group and behaviors in the 50s group (see Table 4).

**Table 4:** The structural model’s coefficients of multigroup SEM.

IV	DV	20–29 years old (n=84)			30–39 years old (n=148)	40–49 years old (n=129)		50 years or older (n=88)	
		β	95% C.I.	β	95% C.I.	β	95% C.I.	β	95% C.I.
Economic	Attitude	0.636***	0.320 – 0.894	0.427***	0.174 – 0.608	0.388***	0.155 – 0.528	0.297*	0.010 – 0.322
Environmental		0.054	-0.107 – 0.174	0.154	-0.023 – 0.191	0.128	-0.022 – 0.168	0.068	-0.060 – 0.110
Interaction		0.377	-0.005 – 0.433	0.428**	0.070 – 0.330	0.424***	0.100 – 0.309	0.250	-0.016 – 0.194
Reputation		-0.157	-0.254 – 0.103	-0.149	-0.195 – 0.057	0.237*	0.020 – 0.176	0.275	-0.006 – 0.213
Economic	Behavior	0.313*	0.034 – 0.648	0.268*	0.049 – 0.516	0.366**	0.168 – 0.721	0.352*	0.044 – 0.372
Environmental		0.176*	0.005 – 0.247	-0.012	-0.116 – 0.101	-0.096	-0.201 – 0.05	0.319**	0.033 – 0.219
Interaction		-0.094	-0.259 – 0.138	0.015	-0.128 – 0.143	-0.172	-0.262 – 0.033	-0.245	-0.199 – 0.015
Reputation		0.140	-0.078 – 0.232	0.133	-0.059 – 0.200	0.037	-0.083 – 0.126	0.280*	0.001 – 0.222
Attitude		0.514**	0.215 – 0.957	0.494***	0.281 – 0.854	0.661***	0.425 – 1.396	0.133	-0.139 – 0.420
**IV**	**DV**	**Married (n=235)**				**Single (n=214)**			
		**β**		**95% C.I.**		**β**		**95% C.I.**	
Economic	Attitude	0.455***		0.241 – 0.556		0.413***		0.193 – 0.492	
Environmental		0.104		-0.020 – 0.133		0.157*		0.004 – 0.171	
Interaction		0.440***		0.113 – 0.299		0.178*		0.000 – 0.175	
Reputation		-0.087		-0.116 – 0.040		0.299**		0.055 – 0.220	
Economic	Behavior	0.480***		0.323 – 0.710		0.169		-0.004 – 0.301	
Environmental		-0.046		-0.112 – 0.050		0.117		-0.012 – 0.151	
Interaction		-0.027		-0.117 – 0.086		-0.179*		-0.180 – -0.006	
Reputation		0.142		-0.008 – 0.160		0.150		-0.009 – 0.155	
Attitude		0.374***		0.229 – 0.690		0.563***		0.350 – 0.836	

Notes. This table presents the results of two separate multiple group SEM analyses by age (upper) and marital status (lower), respectively. *p < 0.05, **p < 0.01, ***p < 0.001. The values are standardized coefficients, and the values in parentheses are standard errors. IV and DV refer to independent and dependent variables, respectively. For gender, income, education, and past experience, SEM analysis was not conducted as measurement invariance assumptions were not met.

In comparison by marital status, economic and interaction motivations significantly influenced attitudes in both married and unmarried groups. Environmental and reputation motivations significantly influenced attitudes only among unmarried individuals. Among the behavioral outcomes, interaction motivations significantly affected behavior only among unmarried respondents, whereas economic motivations did so only among married individuals. Lastly, the effect of attitude on behavior was significant in both groups, with a notably stronger influence observed among the unmarried group (see Table 4).

## 5. Discussion: decoding the dynamics of LDSP engagement

Digital platforms have expanded opportunities once limited to established communities with pre-existing mutual trust and familiarity among members, by offering a “new form of coordination and efficiency” [60, p. 93]. Karrot Market exemplifies this innovation in neighborhood-based digital commerce. Using the case of Karrot Market, this study examined user motivations for this LDSP approach, aiming to enrich the existing literature.

### 5.1. Theoretical and empirical implications of the findings

This section provides interpretations of our findings through the theoretical lenses that we adopted in the research model. Notably, according to our SEM analysis, economic incentives served as the most influential driver of LDSP use, exhibiting the strongest direct and indirect effects among all motivations. Reputation motivations also directly influenced LDSP engagement, while environmental and social interaction motivations affected engagement only indirectly through attitudes.

As discussed in the literature, SDT points out that motivations exist along a continuum from extrinsic (controlled) to intrinsic (autonomous) forms. In our model, economic motivation aligns with external regulation, as users are motivated by tangible rewards in the form of cost savings. Reputation motivation aligns with introjected regulation, suggesting that users engage with LDSPs partly due to internal pressures to maintain self-worth or seek social approval through the platform’s reputation evaluation system. Although it is relatively more autonomous, it still reflects actions driven by internalized social expectations rather than purely intrinsic enjoyment. In comparison, environmental motivations integrated personal sustainability values but were the least influential, suggesting that users’ sustainability concerns alone may not be strong enough to drive immediate LDSP use without additional incentives. Interaction motivation, which is closest to intrinsic motivation for its association with inherent enjoyment and relatedness, did not directly influence LDSP use, suggesting that intrinsic enjoyment alone may not drive participation unless it translates into more instrumental benefits. These findings reaffirm TRA’s proposition that attitudes mediate the relationship between motivations and behavioral intentions.

Our results show some discrepancies from those of key prior studies. Hamari et al. [26] found that enjoyment (which is comparable to social interaction motivation in our study) was the most influential factor, followed by economic and environmental motivations. Ek Styvén and Mariani [25] identified environmental motivations as the most influential driver of DSP engagement. These differences may stem from the distinct focuses and purposes of the DSPs examined in those studies. While Hamari et al. [26] and Ek Styvén and Mariani [25] studied a DSP builder and a used clothing DSP, respectively, our study focused on a specific LDSP and its users with experience in secondhand purchasing.

Another explanation lies in Karrot Market’s branding and user perceptions. Unlike platforms explicitly promoting sustainability, Karrot emphasizes connecting with neighbors, offering features beyond secondhand trade, such as community activity posts. Consequently, users may engage without consciously associating their actions with pro-environmental behavior. Moreover, users may not perceive the environmental benefits of buying or selling used items. For instance, buying a used t-shirt from a neighbor may not create an immediate sense of saved resources, energy, or water. These limited cognitive connections to environmental benefits may diminish the role of environmental motivations in LDSP engagement.

We found that positive attitudes toward DSPs enabled all motivations to influence DSP participation, serving as a mediating mechanism. This result is especially important for environmental and social interaction motivations, which did not directly drive behavior but were translated into behaviors only through positive attitudes. This finding aligns well with TRA, which posits that attitudes mediate the relationship between underlying beliefs/motivations and behavioral intention. Different from the findings by [26] and [34], interaction motivations did not directly drive decisions about continued purchasing used goods through Karrot. Instead, social interaction drivers exhibited the second most influential effect on LDSP engagement via attitudes. On the other hand, environmental motivations showed weaker indirect effects, suggesting that Karrot Market may not sufficiently appeal to users with the value of circular consumption. Overall, the identified interplay between motivations and attitudes aligns with the TRA framework, which underscores that motivations alone are insufficient to predict behavior unless they are internalized as positive attitudes toward buying secondhand goods through LDSPs.

Distinct motivational patterns for LDSP engagement were observed across different socioeconomic groups. Environmental motivations increased behavioral intention among users in their 20s or over 50s as well, differing from [25], where age did not moderate the association between environmental motivations and attitudes. Reputation motivations had greater significance for the oldest in our study, contrasting with conventional findings that younger populations are more reputation-sensitive on social media [61,62]. This could reflect the nature of LDSPs, different from that of regular social media platforms. While Karrot discloses some user information and allows social interactions like social media, communication topics, and the disclosed information are generally limited to trading via the LDSP. A possible connection between the high reputation and successful trading might have mattered to older users more.

Marital status also shaped these associations. Married users tended to prioritize economic and interaction motivations, aligning with previous findings that married individuals are more likely to engage in green purchasing behaviors [63]. Although LDSPs are designed to foster local interactions through features such as neighborhood bulletins, in practice, these interactions are primarily limited to online chatting, mainly for negotiating prices and arranging exchanges. As a result, physical encounters are often brief and transactional. This suggests that online interaction alone, when limited to functional exchanges for secondhand purchases, may not be sufficient to promote sustained use of the platform, particularly for singles. Additionally, our analysis revealed that environmental motivations positively influenced attitudes among users who relied solely on Karrot, while economic and social motivations were more prominent for those engaged in multiple DSPs. As Böcker and Meelen [23] pointed out, user motivations can vary by DSP type—for example, environmental motivations were more potent for ride-sharing DSPs in their study. Our findings add empirical evidence that environmental motivations support LDSP participation, although their impact occurs indirectly through positive attitudes.

### 5.2. Policy implications for catalyzing the local circular economy

Our findings highlight the potential of economic incentives in promoting sustainable consumption, given that secondhand purchases can contribute to the circular economy by extending the lifetime of goods and increasing the utilization rate by selling and buying the underutilized resources that would otherwise require significant storage space. The influential role of economic motivations, as confirmed in our analysis, can engage a broad spectrum of users, including those not initially motivated by environmental concerns, thereby encouraging wider participation in circular consumption.

However, this finding also reveals a critical policy challenge: while economic motives are effective entry points, fostering stronger environmental motivations among DSP users is essential to mitigate rebound effects such as over-consumption [64], particularly in the context of non-essential goods [28]. Policymakers should consider these multifaceted factors behind LDSP participation—including resale, repurchase, and recycling—as part of future policies and interventions, as underscored by van Bussel et al. [65].

Local circular economy activities can be expanded and adapted in different contexts, provided that socio-cultural factors are carefully considered. For example, Karrot Market has been introduced in Western countries such as the U.S. and Canada, with platform features localized to fit community norms and user behaviors [44]. As the United Nations’ Sustainable Development Goals (SDGs) highlight these efforts—particularly under SDG 11 (Sustainable Cities and Communities) and SDG 12 (Responsible Consumption and Production)—LDSPs could be more systematically integrated into national and subnational sustainability policies through partnerships with the private sector and civil society.

For example, an innovative intervention could involve the development of public, non-profit LDSPs that prioritize environmental outcomes. To make the environmental benefits of circular economy activities more tangible and meaningful to users, platforms could implement a point-based incentive system—e.g., awarding ‘eco-points’ to sellers and buyers based on estimated greenhouse gas emissions reduced through secondhand trading. These environmental savings expressed in intuitive metrics (e.g., equivalent to the electricity consumption of a four-person household for one day) could be visually displayed alongside item listings and even linked to dynamic pricing schemes that reflect ecological impact. Seoul’s existing eco-point system provides a relevant precedent, where citizens earn points for conserving electricity or reducing car use [66]. These points can be redeemed for purchases at local shops or even used to pay city taxes. If such systems were integrated into LDSPs, users could earn and spend eco-points directly on secondhand goods, thereby linking environmental behavior with visible benefits.

Another potential policy approach is to recognize and certify businesses in the secondhand commerce sector for their environmental contributions. For example, a certification program—similar to the B Corp Certification—could be developed to highlight best practices and benchmark sustainability performance. This could include a localized green corporate certification for businesses engaged in circular economy models. Such certification schemes could also support local governments in identifying eligible partners for public awareness campaigns or sustainability education efforts.

Finally, incorporating the diversity of user motivations, especially across different generational groups and household types, into policy interventions can help enhance the effectiveness of communication strategies and public messaging for sustainable consumption. For instance, individuals in their 20s or those in their 50s or above may be more responsive to campaigns promoting sustainability, whereas middle-aged users may prioritize economic practicality. Household composition can also play a role: families, especially those with children, may prioritize affordability and seek more secondhand items that require frequent replacement, such as children’s clothes and toys. LDSP design and policy interventions could therefore benefit from understanding these demographic-specific tendencies to promote long-term engagement across diverse user groups.

### 5.3. Limitations and future research

We identify key limitations of our study, which suggest future research directions. First, our survey did not differentiate user motivations, attitudes, and behaviors toward purchasing necessities versus non-necessities. Additional research is necessary to assess potential over-consumerism in DSP engagement and its diverse patterns across different socioeconomic groups. Applying qualitative or mixed methods approaches could be beneficial in this future work. Second, although our study contributes by focusing on a non-Western context—an area underrepresented in previous research—it is limited to the case of South Korea. As such, it does not capture the broader diversity of cultural norms and practices related to secondhand consumption. Future research should conduct cross-cultural comparisons to examine how user motivations differ across socio-cultural settings. Third, although our dataset included both frequent Karrot users and those with limited familiarity, we did not distinguish between these user groups in the analysis. This may have introduced potential bias, as varying levels of platform experience could reflect heterogeneity in motivations and attitudes toward LDSPs, and therefore participation behavior. Future research should consider stratifying samples based on user familiarity or frequency of use to reduce such bias and capture user-specific dynamics more accurately.

## 6. Conclusions

This study examined the relationships among LDSP participants’ motivations, attitudes, socioeconomic characteristics, and behavioral intentions regarding secondhand purchases, using Karrot Market in South Korea as an empirical case. The platform’s local-centric design offers a unique DSP innovation, facilitating local circular consumption by enabling neighborhood-based exchanges of goods, thereby revitalizing interaction among residents. Users and their community can benefit in multiple aspects, from economic gains (selling and buying reasonably priced pre-owned items) to social and environmental benefits (such as trading within walking distance and extending product lifespans).

Our findings highlight the need to develop consumption cultures and platform features that promote reuse by addressing users’ multidimensional motivations. While relatively extrinsic motivations, including economic and reputation motivations, directly affected users' intentions to continue using the platform, economic, environmental, and interaction motivations were translated into behaviors through attitudes toward Karrot. These results underscore the central role of attitudes as mediators and the need for platform and policy strategies that cultivate positive perceptions of secondhand consumption, thereby fostering more environmental motivations. Moreover, our multigroup analysis revealed that motivational pathways vary by demographic characteristics, such as age and marital status, offering insights for more targeted engagement strategies within the circular economy at the local scale.

## Supporting information

S1 Fig Z-score distribution by latent variable. (TIFF)

S2 Fig Z-score boxplot by latent variable. (TIFF)

S3 Fig Descriptive statistics of demographic variables. (TIFF)

S4 Fig Scree plot of Eigenvalues. (TIFF)

S5 Fig Factor loadings by item (Exploratory factor analysis) (TIFF)

S1 Table Summary of key papers on motivations for DSPs. (PDF)

S2 Table Discriminant validity. (PDF)

S3 Table Comparison results using chi-square test. (PDF)

S1 File Survey questionnaires. (PDF)
