# Supplementary figures and images for: Unraveling participant motivation dynamics in local-centric secondhand digital sharing platforms
Source: PLoS One. 2025 Dec 26;20(12):e0337603. doi: 10.1371/journal.pone.0337603 (PMC12742730; doi:10.1371/journal.pone.0337603)

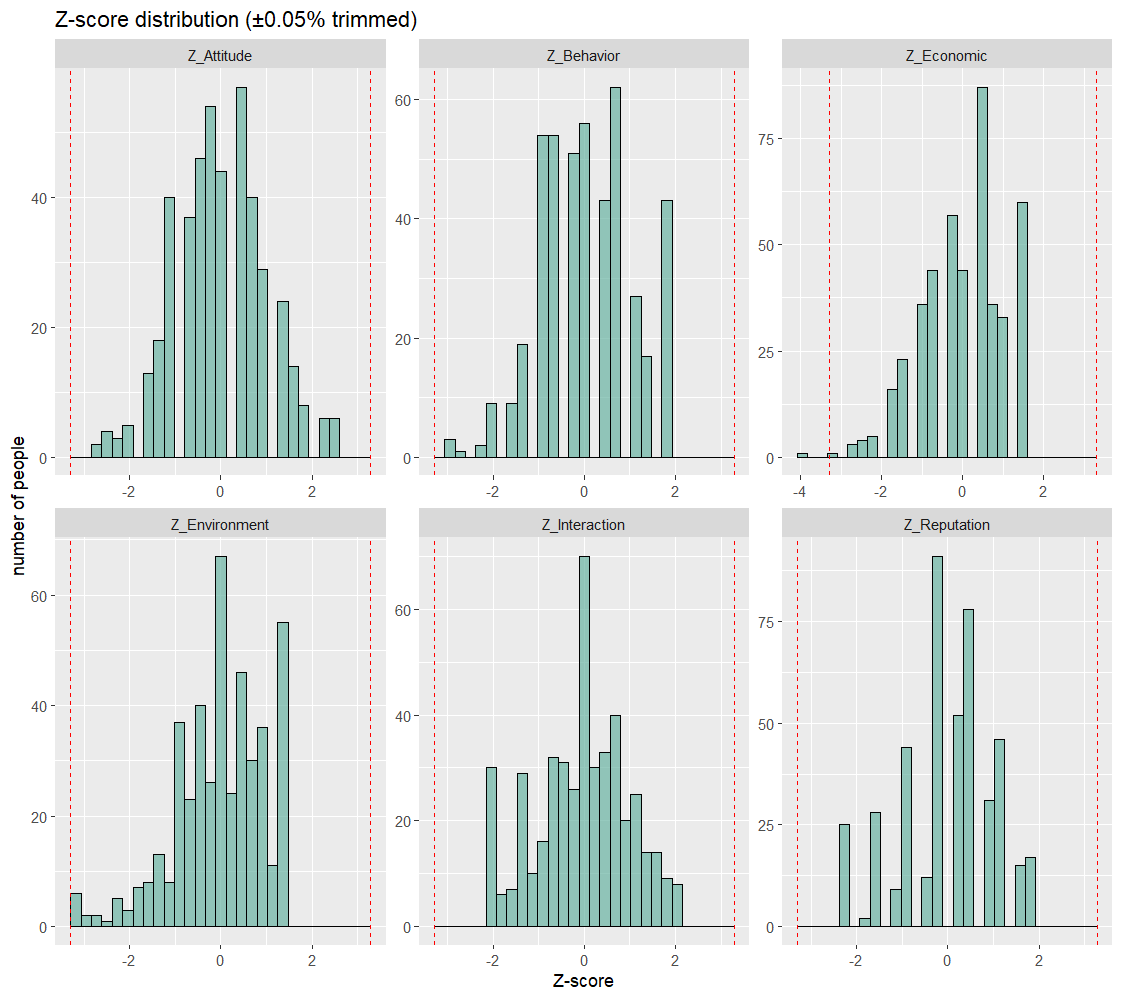

Supplement: S1 Fig — (TIFF) [file pone.0337603.s001.tiff]

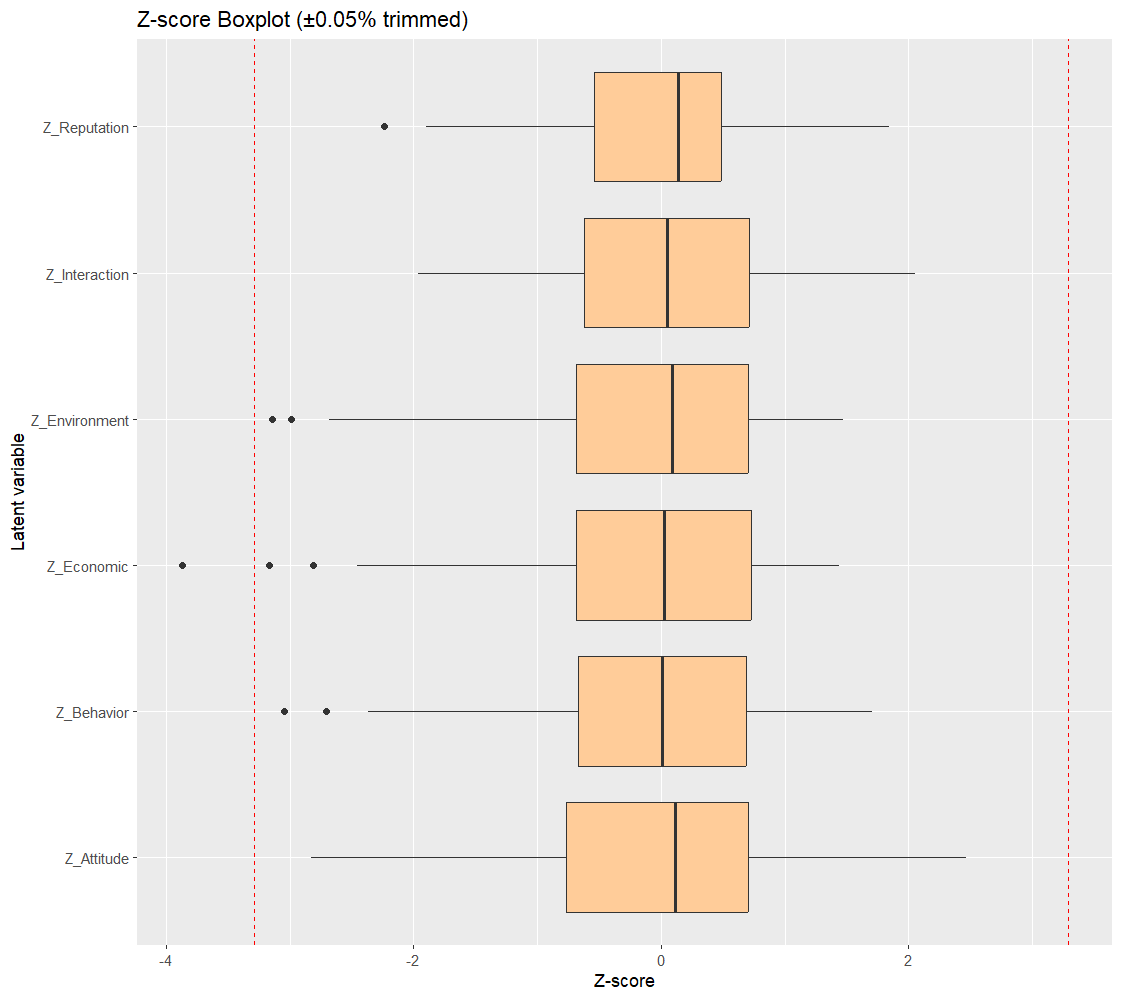

Supplement: S2 Fig — (TIFF) [file pone.0337603.s002.tiff]

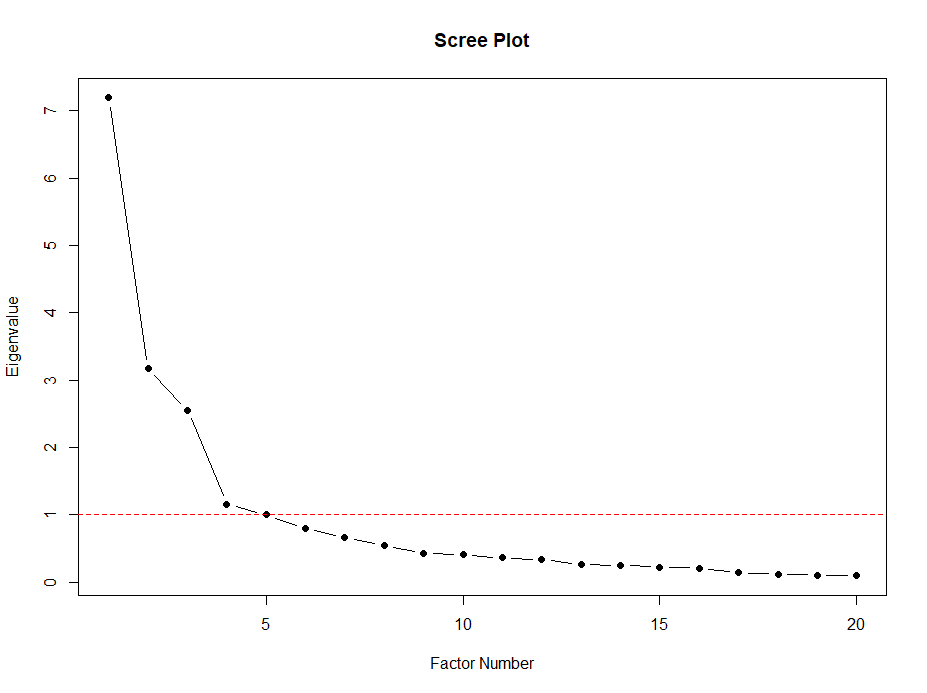

Supplement: S4 Fig — (TIFF) [file pone.0337603.s004.tiff]

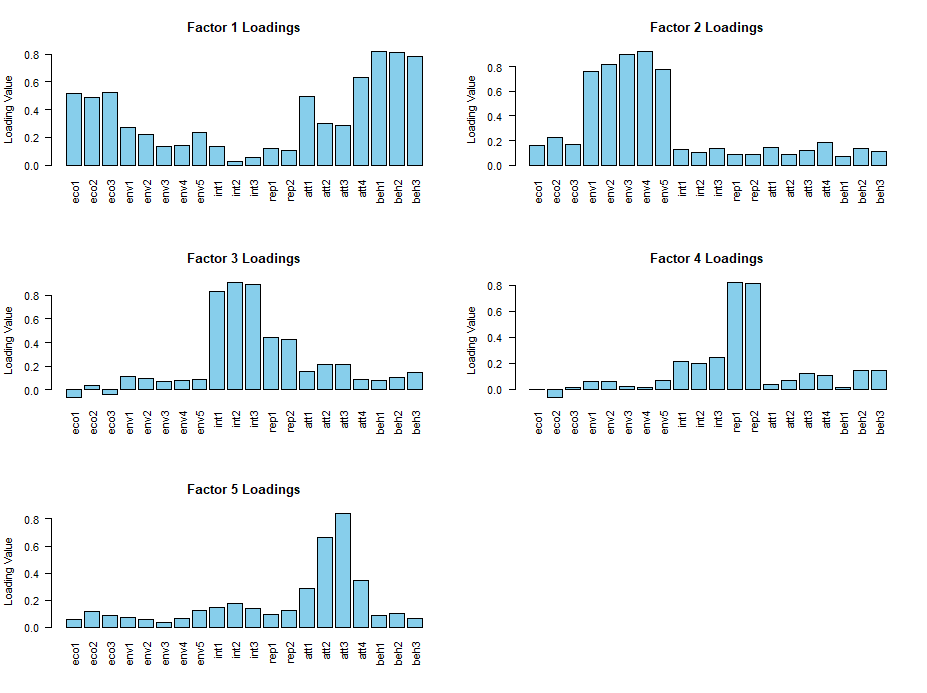

Supplement: S5 Fig — (TIFF) [file pone.0337603.s005.tiff]
