# Supplementary material for: Unraveling participant motivation dynamics in local-centric secondhand digital sharing platforms
Source: PLoS One. 2025 Dec 26;20(12):e0337603. doi: 10.1371/journal.pone.0337603 (PMC12742730; doi:10.1371/journal.pone.0337603)
Supplement: S1 Table — (PDF) [file pone.0337603.s006.pdf]

S1 Table. Summary of key papers on motivations for DSPs.

Ref.	DSP(s) studied	Survey sample size (location)	Theoretical background(s)	Motivations included in the analysis	Quantitative analysis method
[23]	Car, ride, accommodation, tool, and meal sharing	1330 (the Netherlands)	• Three pillars of sustainability	• Economic<br>• Social<br>• Environmental	Ordered logit
[24]	Airbnb (property rentals), Blablacar (car sharing), Shpock (classified ads)	146 (Europe and US)	• Rational choice theory<br>• Transaction cost theory<br>• Customer value framework	• Perceived value co-creation processes<br>• Perceived value proposition design<br>• Perceived value capture mechanisms	SEM
[25]	eBay, Facebook, Gumtree, Depop, Amazon, and Shpock	412 (UK)	• TRA/TPB	• Perceived sustainability<br>• Economic motivations<br>• Distance from the consumption system	SEM
[26]	Sharetribe	168 (Worldwide) (Platform based in Finland)	• SDT	• Intrinsic<br>o Enjoyment<br>o Sustainability<br>• Extrinsic<br>o Economic benefits<br>o Reputation	SEM
[27]	DSP within a university community (hypothetical)	325 (Sweden)	• TPB	• Social Experience<br>• Trust in Others<br>• Ecological Sustainability<br>• Sense of Belonging<br>• Familiarity<br>• Financial Benefits<br>• Variety	Multi-linear regression
[28]	Leboncoin	541 (France)	• Self-licensing theory	• Materialism<br>• Environmental consciousness	Path analysis
[30]	Airbnb	468 (Turkey)	• TPB	• Price value<br>• Authenticity<br>• Enjoyment<br>• Social interaction<br>• Home benefits<br>• Novelty<br>• Sharing economy ethos	SEM
[31]	Ridesharing	485 (Brazil)	• Theoretical review of trust in the sharing economy	• Economic reward<br>• Perceived sustainability<br>• Enjoyment<br>• Platform quality<br>• Security risk	Linear regression
[32]	Hospitality businesses	418 (South Korea)	• Social cognitive theory<br>• Value-based adoption theory	• Social interaction<br>• Social presence<br>• Sustainability<br>• Community belonging<br>• Trust<br>• Price<br>• Familiarity<br>• Accessibility	SEM
[34]	Sharing economy (not specified)	104 (Turkey and Mongolia)	• Followed [26]	• Enjoyment<br>• Economic benefit<br>• Sustainability<br>• Reputation	Linear regression

[41]	Bicycle sharing	373 (China)	• SDT	• Intrinsic (Enjoyment)<br>○ Sustainability<br>○ Sense of belonging<br>○ Trust<br>• Extrinsic (Perceived usefulness)<br>○ Economic benefits<br>○ Convenience	SEM
