# Supplementary material for: Unraveling participant motivation dynamics in local-centric secondhand digital sharing platforms
Source: PLoS One. 2025 Dec 26;20(12):e0337603. doi: 10.1371/journal.pone.0337603 (PMC12742730; doi:10.1371/journal.pone.0337603)
Supplement: S2 Table — (PDF) [file pone.0337603.s007.pdf]

S2 Table. Discriminant validity.

	Economic motivations	Environmental motivations	Interaction motivations	Reputation motivations	Attitude	Behavior
Economic motivations	1.000					
Environmental motivations	.403	1.000				
Interaction motivations	.063	.265	1.000			
Reputation motivations	.100	.218	.681	1.000		
Attitude	.528	.401	.446	.394	1.000	
Behavior	.610	.366	.253	.313	.681	1.000
Sqrt. AVE	.720	.862	.925	.941	.715	.843
Discriminant validity	Y	Y	Y	Y	Y	Y

Note. ‘Y’ in the last row indicates that discriminant validity is confirmed.
