# Supplementary material for: Unraveling participant motivation dynamics in local-centric secondhand digital sharing platforms
Source: PLoS One. 2025 Dec 26;20(12):e0337603. doi: 10.1371/journal.pone.0337603 (PMC12742730; doi:10.1371/journal.pone.0337603)
Supplement: S3 Table — (PDF) [file pone.0337603.s008.pdf]

S3 Table. Comparison results using chi-square test.

			AIC	BIC	Chisq	Chisq diff	Pr (> Chisq)
Sex Group	UM vs CMM	UM	23571	24212	688.16		
		CMM	23559	24142	704.30	16.140	0.305
	CMM vs CSM	CMM	23559	24142	704.30		
		CSM	23545	24091	708.22	3.927	0.916
Age Group	UM vs CMM	UM	23627	24908	1105.8		
		CMM	23584	24693	1147.1	41.372	0.498
	CMM vs CSM	CMM	23584	24693	1147.1		
		CSM	23571	24569	1188.5	41.314	0.038*
Income Group	UM vs CMM	UM	23862	25784	1644.7		
		CMM	23807	25442	1730.5	85.803	0.096
	CMM vs CSM	CMM	23807	25442	1730.5		
		CSM	23778	25227	1790.7	60.136	0.065
Marriage Group	UM vs CMM	UM	23604	24244	675.58		
		CMM	23589	24172	688.70	13.117	0.517
	CMM vs CSM	CMM	23589	24172	688.70		
		CSM	23589	24135	706.62	17.918	0.036*
Experience Group	UM vs CMM	UM	23609	24249	679.21		
		CMM	23600	24183	698.16	18.952	0.167
	CMM vs CSM	CMM	23600	24183	698.16		
		CSM	23598	24144	714.63	16.468	0.057

Note. To test for group differences in structural relationships, we employed a multigroup structural equation modeling (SEM) approach involving three nested models: the Unconstrained Measurement (UM) model, the Constrained Measurement Model (CMM), and the Constrained Structural Model (CSM). First, we compared the UM and CMM models using chi-square difference testing to evaluate measurement invariance. In the UM model, all parameters were freely estimated across groups, while in the CMM, factor loadings were constrained to be equal across groups. A non-significant chi-square difference supports metric invariance, indicating that latent constructs are measured equivalently across groups. This provides a valid basis for comparing structural paths. Next, we compared the CMM and CSM models to test structural invariance. The CSM further constrains the structural path coefficients to be equal across groups. If the chi-square difference between the CMM and CSM is significant, this indicates that structural relationships among latent variables differ across groups.
