# Supplementary material for: Unraveling participant motivation dynamics in local-centric secondhand digital sharing platforms
Source: PLoS One. 2025 Dec 26;20(12):e0337603. doi: 10.1371/journal.pone.0337603 (PMC12742730; doi:10.1371/journal.pone.0337603)
Supplement: S1 File — (PDF) [file pone.0337603.s009.pdf]

S1 File. Survey questionnaires.

Household Characteristics

1. What is your gender?
2. What is your age?
3. What is your place of residence?
4. Please select all the apps you have used from the list below.
5. Have you ever bought or sold items on the secondhand trading platform Karrot Market?
6. Have you ever bought second-hand goods (excluding unopened new items) via Karrot Market?
7. Are you currently married?
8. Including yourself, how many people live in your household at the same address?
9. What is your highest level of education?
10. What is your household's average monthly income after tax?
11. What is your current occupation?
12. Before using Karrot Market, have you traded second-hand goods through other platforms (e.g., Jungonara, eBay)?
13. How frequently did you purchase second-hand goods via Karrot Market in the past year (2021)?
14. Compared to before COVID-19 (2019), has your average annual purchase frequency of second-hand goods on Karrot Market increased during 2020–2021?
15. In the past year (2021), how often did you make face-to-face purchases when using Karrot Market?
16. In the past year (2021), how often did you make purchases via parcel delivery when using Karrot Market?
17. If there were other methods you used to purchase second-hand goods via Karrot Market, please specify.
18. Based on your transaction history on the Karrot Market app, how many second-hand purchases did you make in the past year (2021)?
19. Please select all types of second-hand goods you purchased via Karrot Market in the past year (2021).

20. I am still actively using the second-hand goods I purchased via Karrot Market in the past year (2021).
21. Please select the types of second-hand goods you are still actively using.

The following questions ask about how you disposed of secondhand items you purchased on Karrot Market within the past year (2021) that you no longer use or do not plan to use in the future. (If multiple items come to mind, please respond based on the main method of disposal you used.)

22. I plan to resell the secondhand items I will not use through online platforms such as Karrot Market.
23. I plan to give away unused second-hand goods to acquaintances or others.
24. I plan to dispose of unused second-hand goods.
25. Have you ever used any other digital sharing platforms besides Karrot Market (e.g., car sharing)?
26. Which digital sharing platforms (besides Karrot Market) have you used?
27. Overall, how satisfied are you with Karrot Market?
28. Among the following second-hand platforms, which one do you prefer most?
29. Do you plan to continue using Karrot Market in the future?
30. Would you recommend Karrot Market to others?

The following questions are about your motivations for purchasing secondhand items on Karrot Market. Please indicate the extent to which you agree with each statement.

Economic Motivations

31. I can buy more items within my limited budget.
32. I can afford to buy more of what I need.
33. I want to save money.
34. I don’t want to pay more just because an item is new.
35. I want to buy items at a fair price.

Environmental Motivations

36. I want to reduce resource consumption (related to the production and sale of goods) by buying fewer new products.
37. I want to reduce energy consumption (related to the production and sale of goods) by buying fewer new products.
38. I want to reduce waste (such as plastic and packaging) by buying fewer new products.
39. I want to protect the environment by buying fewer new products.
40. I want to practice sustainable consumption by buying fewer new products.

Interaction Motivations

41. It is fun to communicate (chat) with local neighbors while buying used items on Karrot.
42. I am interested in communicating (chatting) with local neighbors while buying used items on Karrot.
43. I enjoy communicating (chatting) with local neighbors while buying used items on Karrot.

Reputation Motivations

44. It is fun to manage my ‘manner temperature’ (reputation score) while buying used items on Karrot.
45. I am interested in managing my ‘manner temperature’ (reputation score) while buying used items on Karrot.
46. I enjoy managing my ‘manner temperature’ (reputation score) while buying used items on Karrot.

Attitudes

47. I find buying used items on Karrot convenient.
48. Buying used items on Karrot feels safe.
49. Buying used items on Karrot feels reliable.
50. Buying used items on Karrot is a wise decision.

Behaviors

51. I intend to continue to buy used items on Karrot frequently.
52. If possible, I intend to buy used items on Karrot frequently.
53. I am likely to buy used items on Karrot frequently.
